# Long Non‐Coding RNAs: Key Regulators of Economic Traits in Livestock From Mechanisms to Breeding Applications

**DOI:** 10.1002/vms3.71292

**Published:** 2026-10-21

**Authors:** Keyvan Khani, Mohammad Taher Harkinezhad, Rahimeh Sepehri

**Affiliations:** ^1^ Department of Animal Science, Faculty of Agriculture University of Zanjan Zanjan Iran

**Keywords:** animal breeding, circRNA, CRISPR‐Cas13, economic trait, lncRNA

## Abstract

Long non‐coding RNAs (lncRNAs) have emerged as critical regulators of economically important traits in livestock, including growth, reproduction, disease resistance and product quality. Unlike protein‐coding genes, lncRNAs orchestrate gene expression at epigenetic, transcriptional and post‐transcriptional levels, acting as molecular switches, scaffolds, decoys, and competing endogenous RNAs (ceRNAs). This review synthesises current knowledge on key lncRNA mechanisms such as ceRNA networks, liquid–liquid phase separation (LLPS) and RNA‐targeting clustered regularly interspaced short palindromic repeats (CRISPR‐Cas13) screening alongside trait‐specific case studies in muscle hypertrophy (Callipyge locus), embryonic development (lnc_2217 in goats) and fecundity (ceRNA networks in sheep and chickens). We highlight the integration of lncRNAs with Genome Wide Association Studies (GWAS) data, where over 90% of trait‐associated variants reside in non‐coding regions, and propose a practical roadmap for functional validation and incorporation of lncRNAs into genomic selection and breeding programs. Harnessing lncRNA biology supported by recent functional annotation of animal genomes (FAANG) epigenomic maps and CRISPR‐Cas13 applications in livestock, researchers will accelerate genetic gain in growth rate, marbling score, litter size and disease resilience, marking a paradigm shift toward precision livestock breeding.

## Graphical abstract

Long non‐coding RNAs (lncRNAs) act as key regulators of economically important traits in livestock through diverse mechanisms including ceRNA networks, epigenetic regulation (X‐inactivation and imprinting) and liquid–liquid phase separation (LLPS). Integration of lncRNA biology with GWAS, CRISPR‐Cas13 RNA targeting and genome analysis provides a practical roadmap for improving meat quality, growth rate, fertility and disease resistance, paving the way toward precision livestock breeding.

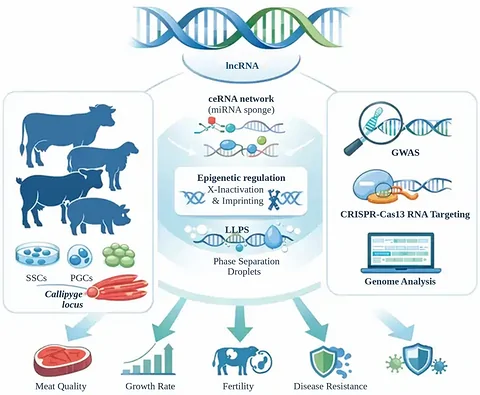

## 1 Introduction

Recent advances in transcriptomics have revealed that the vast majority of the mammalian genome is transcribed into non‐coding RNAs (ncRNAs), many of which play critical regulatory roles in development, physiology and economically important traits in livestock (Mercer et al. 2009). It is now well established that protein‐coding genes account for less than 2% of the genome, while the remaining portion gives rise to a diverse array of non‐coding transcripts, including thousands of long non‐coding RNAs (lncRNAs) (Mercer et al. 2009; Kosinska‐Selbi et al. 2020). The generation of such a large population of lncRNA transcripts indicates that RNAs play a major and important regulatory role in various biological processes and their misregulation contributes to various human diseases, including cancer (Esteller 2011). In recent years, a large number of nuclear lncRNAs have been discovered. These lncRNAs play diverse roles in the nucleus through different mechanisms; for example, nuclear lncRNAs control the epigenetic state of specific genes, participate in transcriptional regulation and are involved in alternative splicing (Bernard et al. 2010; Wilusz 2016). Recent transcriptomic studies indicate that a substantial fraction of the mammalian transcriptome consists of ncRNAs, highlighting their pervasive regulatory functions. Unlike protein‐coding mRNAs, lncRNAs function primarily as regulators of gene expression at multiple levels: epigenetic, transcriptional and post‐transcriptional (Liang et al. 2024). They act as molecular switches, scaffolds, decoys and guides, thereby influencing key biological processes. Based on their genomic location, most lncRNAs can be characterised as large intergenic non‐coding RNAs, natural antisense transcripts (NATs), pseudogenes, long intronic lncRNAs and other divergent transcripts (Dykes & Emanueli 2017).

LncRNAs are quite complicated and share many features with protein‐coding RNAs. In the nucleus, they occupy the chromatin fraction. The study reveals that 17% of lncRNAs to 15% of mRNAs are enriched in the nucleus, while 4%–26% are enriched in the cytoplasm (Rashid et al. 2016). In the cytoplasm, thousands of cytoplasmic lncRNAs have been discovered in the last decade, indicating their importance in various cellular activities (Rashid et al. 2016). In cells, several methods such as genome‐wide chromatin immunoprecipitation‐sequencing (ChIP‐seq) and RNA immunoprecipitation followed by sequencing (RIP‐seq) have been developed to study the genomic localisation of lncRNAs and their interacting proteins (Ransohoff et al. 2018). In the nucleus, lncRNAs often function in cis to modify local gene transcription. Cis‐acting lncRNAs are often expressed at low abundance, with only a few copies per cell, and their importance may be dismissed by high‐throughput studies. In addition, in the nucleus, some other lncRNAs act in trans to regulate the expression of loci distant from their sites of synthesis in multiple chromosomes (Morlando and Fatica 2018). Genome‐wide approaches have shown that mutations in regulatory regions, which alter enhancer and promoter sequences or their chromatin state, lead to aberrant lncRNA expression in tumours compared with normal tissues (Dykes and Emanueli 2017). Importantly, genome wide association studies (GWAS) have shown that more than 90% of trait‐associated variants lie within non‐coding genomic regions (Peña‐Martínez and Rodríguez‐Martínez 2024). This strongly suggests that regulatory elements, including lncRNAs, make substantial contributions to phenotypic variation in livestock (Laurent et al. 2014). This review focuses on the biological functions and molecular mechanisms of lncRNAs in livestock species, with particular emphasis on GWAS, epigenetics, skeletal muscle development, disease resistance and reproduction. Furthermore, the development of high‐throughput RNA‐targeting tools, particularly clustered regularly interspaced short palindromic repeats (CRISPR‐Cas13), has opened new avenues for functional interrogation of lncRNAs (Liang et al. 2026). Unlike DNA‐targeting methods (e.g., CRISPR‐Cas9), Cas13 enables direct perturbation of RNA transcripts without altering the underlying genomic DNA, thereby minimising off‐target effects on nearby coding genes (Liang et al. 2026).

A landmark study employed a transcriptome‐scale Cas13 screening approach (CaRPool‐seq) to probe approximately 6200 lncRNAs across five human cell lines (*HAP1, HEK293T, K562, MDA‐MB‐231* and *THP1*), identifying lncRNAs essential for cellular fitness with context‐specific or broader roles, and highlighting their previously underestimated importance in cellular fitness and cancer biology (Liang et al. 2024). Recent RNA‐targeting CRISPR‐Cas13 systems provide a powerful platform for functional interrogation of lncRNAs while avoiding permanent genomic alterations. These approaches have facilitated large‐scale identification of functional lncRNAs and hold considerable promise for livestock genomics (Liang et al. 2026).

These technological advancements are now beginning to be translated into livestock research, paving the way for the systematic discovery of functional lncRNAs that influence economically important traits (such as growth, reproduction, disease resistance and product quality) (Li et al. 2023). Although large‐scale transcriptome‐wide Cas13 screens in livestock species have not yet been reported at the same scale as in human cells, related CRISPR‐based approaches are increasingly applied in animal models to interrogate lncRNA functions during development and in agricultural contexts (Wang et al. 2025). The central argument of this review is that lncRNAs represent a largely untapped regulatory layer that can be systematically converted from descriptive transcriptomic catalogues into actionable breeding tools through three sequential steps: (i) prioritising candidate lncRNAs from GWAS and transcriptome data, (ii) functionally validating their roles using CRISPR‐Cas13 and related RNA‐targeting approaches in livestock primary cells and (iii) incorporating validated lncRNA markers into genomic selection pipelines. In this review, we provide a translational roadmap for animal breeders and geneticists structured around exactly these three steps: how to prioritise lncRNA candidates from GWAS and transcriptome data (Sections 2–3), how to validate their function using CRISPR‐Cas13 in livestock primary cells (Section 3.6) and how to incorporate lncRNA markers into genomic selection pipelines (Section 7) (Figure 1). We also highlight species‐specific resources (databases and expression atlases) and outstanding challenges, including the lack of in vivo functional studies and poor sequence conservation across breeds. Ultimately, our goal is to equip researchers with the knowledge and tools needed to convert lncRNA discoveries into tangible genetic improvements in meat quality, reproductive efficiency and disease resistance in livestock and poultry.

**FIGURE 1:**
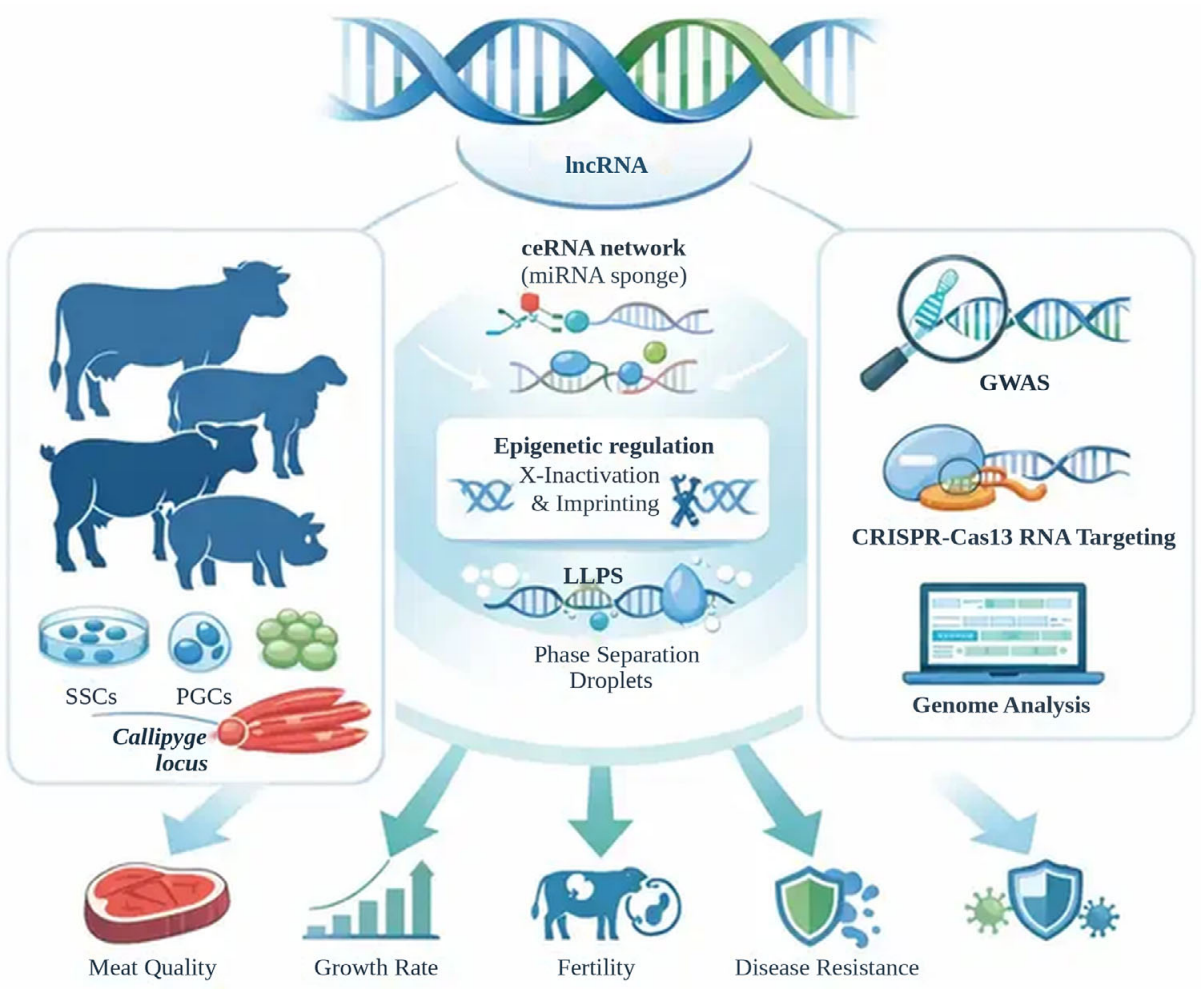
Graphical overview of the regulatory roles of long non‐coding RNAs (lncRNAs) and their potential applications in livestock breeding.

## 2 LncRNA Characteristics

### 2.1 Origins of LncRNA

Most ncRNAs experience minimal evolutionary constraint, resulting in limited sequence conservation across distant species such as mammals and fish. This rapid evolutionary turnover means that many lncRNAs appear and disappear within specific lineages (Ponting et al. 2009). Unlike protein‐coding genes, which typically evolve through gene duplication and sequence divergence, lncRNA genes often arise via distinct mechanisms, including the transformation of protein‐coding sequences into non‐coding ones (Elisaphenko et al. 2008). A well‐known example is the *Xist* gene, which encodes a lncRNA essential for X chromosome inactivation in eutherian mammals. Several exons and promoters of *Xist* originated from remnants of an ancestral protein‐coding gene (*Lnx3*) that accumulated frame‐disrupting mutations during the evolution of placental mammals (Elisaphenko et al. 2008). Such evolutionary processes highlight the dynamic nature of lncRNA origins, contributing to their functional diversity in regulating gene expression across mammalian species, including livestock (Kampa et al. 2004).

### 2.2 Diversity of LncRNA

LncRNAs are broadly classified based on their length into two major groups. NcRNAs shorter than 200 nucleotides (nt) include small RNAs such as microRNAs (miRNAs), small interfering RNAs (siRNAs) and PIWI interacting RNAs (piRNAs), whereas those longer than 200 nt are categorised as lncRNAs (Siomi et al. 2011; Figure 2). Although only a limited number of lncRNAs have been functionally characterised, they are known to regulate gene expression at multiple levels, including transcriptional silencing through chromatin modification and post‐transcriptional processes such as RNA maturation, transport and translation (Wang and Chang 2011). LncRNAs share several features with protein‐coding transcripts, such as epigenetic marks associated with differential expression. Many are intergenic, located outside or non‐overlapping with protein‐coding genes and are often referred to as long intergenic non‐coding RNAs (lincRNAs) (Hangauer et al. 2013). In addition to size‐based classification, the HUGO Gene Nomenclature Committee (HGNC) has categorised lncRNAs into nine subgroups according to their genomic context and relationship with protein‐coding genes (Chodurska and Kunej 2025). The most abundant classes are long intergenic ncRNAs (LINC, approximately 40%), antisense RNAs (approximately 34%) and divergent transcripts (approximately 10.6%) (Figure 2). This standardised classification system greatly facilitates annotation and comparative studies across species. Readers are directed to panels (A–E) of Figure 2 for, respectively: genomic classification of lncRNAs, circular RNA (circRNA) subtypes, mechanistic modes of action, associated physiological functions and representative GO (Gene Ontology) terms, each discussed in the corresponding subsections below.

**FIGURE 2:**
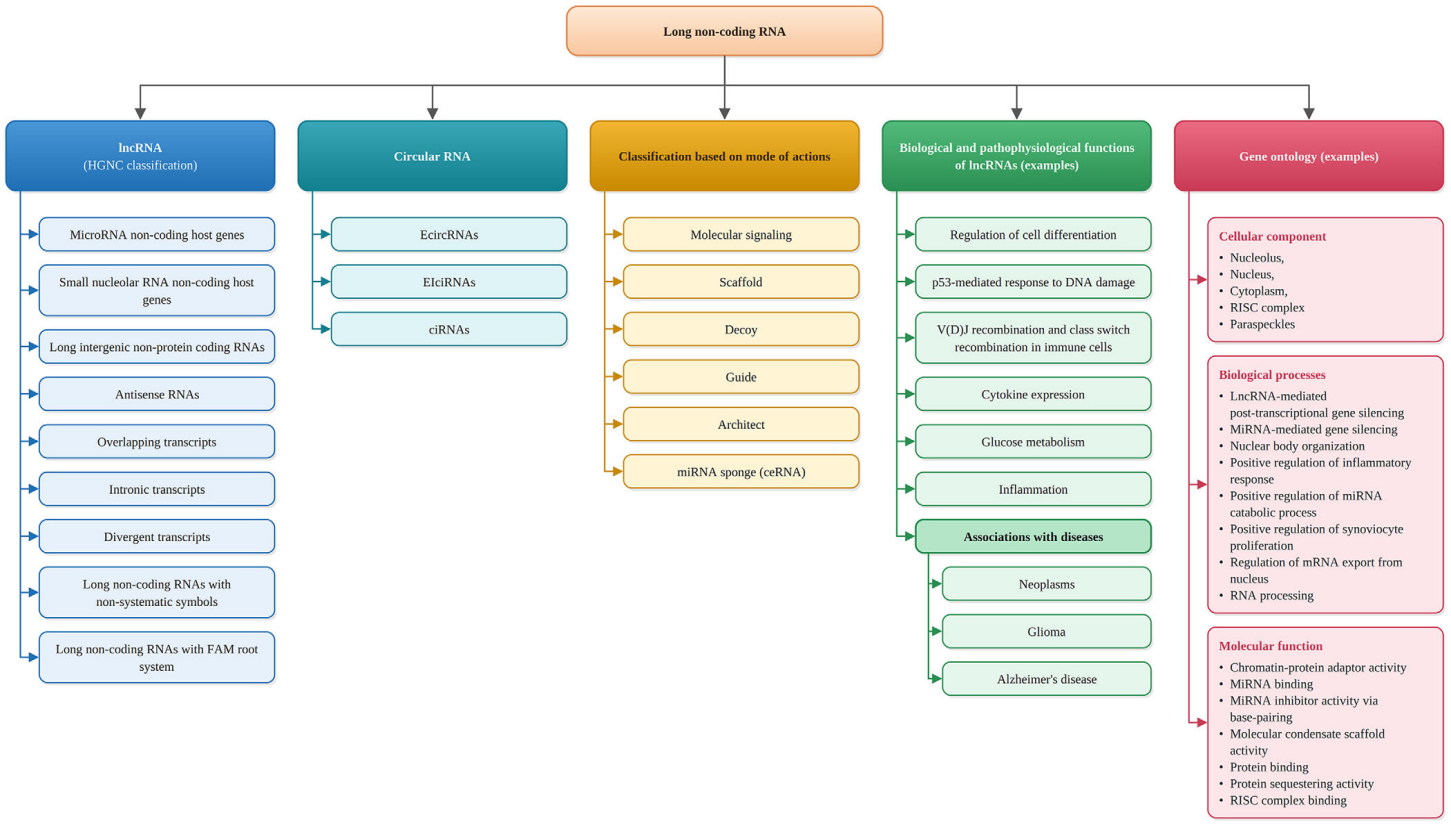
Comprehensive classification and functional landscape of lncRNAs. Figure presents a multi‐dimensional overview of lncRNAs based on genomic classification, structural features, molecular mechanisms and biological functions. (A) Genomic classification: lncRNAs are categorised based on their genomic location relative to protein‐coding genes, including lincRNAs, natural antisense transcripts (NATs), overlapping transcripts, intronic transcripts, divergent transcripts, host genes for small RNAs, pseudogenes and enhancer‐derived RNAs (eRNAs). (B) Circular RNAs (circRNAs): including exonic circRNAs (EicrRNAs), intronic CircRNAs (ciRNAs) and those functioning as miRNA sponges. (C) Modes of action: lncRNAs act as molecular signals, decoys, guides, scaffolds, architects and miRNA sponges (ceRNA), with some also serving as scaffolds for nuclear subcompartment formation such as paraspeckles. (D) Biological functions: lncRNAs are involved in cell differentiation, DNA damage response, immune regulation (V(D)J recombination, class switch recombination and cytokine expression), glucose metabolism and inflammation, and are associated with diseases including neoplasms, glioma and Alzheimer's disease. (E) Gene ontology (GO) terms: selected GO terms illustrate subcellular localisation (nucleolus, nucleus, cytoplasm, paraspeckles and RNA‐induced silencing complex (RISC) complex), biological processes (post‐transcriptional and miRNA‐mediated gene silencing, nuclear body organisation, inflammatory regulation, mRNA export and RNA processing) and molecular functions (chromatin‐protein adaptor activity, miRNA binding/inhibition, protein binding/sequestering and RISC complex binding)(Chodurska and Kunej 2025).

### 2.3 LncRNA Transcription

Early genome‐wide studies revealed pervasive transcription across the mammalian genome. For instance, an Affymetrix tiling array analysis of human chromosomes 21 and 22 showed that 94% of detected transcripts originated from regions outside known exons (Kapranov et al. 2002). Subsequent reports estimated that nearly 49% of transcribed nucleotides lie outside annotated genes (Kampa et al. 2004). More comprehensive analyses using cap analysis of gene expression (CAGE) further demonstrated that approximately 63% of the mammalian genome is transcribed, highlighting the extensive transcriptional activity in both coding and non‐coding regions (Kampa et al. 2004). These findings underscore that lncRNA transcription is a widespread phenomenon, contributing significantly to the complexity of gene regulation in mammals, including livestock species.

### 2.4 LncRNA Biology: Mechanisms of Action

Recent studies have shown that lncRNAs play diverse and multifaceted roles in cellular metabolism, development and gene regulation. These roles include genomic imprinting, chromatin modification, transcription, splicing, cell cycle control, mRNA decay and translation (Zhu et al. 2013). LncRNAs regulate gene expression at three primary levels: epigenetic, transcriptional and post‐transcriptional (Hung et al. 2011; Kretz et al. 2013). They are abundant in the genome and generally lack functional open reading frames (ORFs), consistent with their non‐coding nature.

LncRNAs exert their regulatory functions through six major modes of action. Rather than operating as isolated mechanisms, these modes frequently intersect in a context‐dependent manner, allowing lncRNAs to fine‐tune gene expression during dynamic processes such as myogenesis, folliculogenesis and immune activation.

#### 2.4.1 Molecular Signals

LncRNAs respond to specific cellular stimuli and recruit chromatin‐modifying enzymes to target genes (Böhmdorfer and Wierzbicki 2015). In livestock, this mode is exemplified by stress‐responsive lncRNAs in bovine mammary tissue that rapidly recruit histone modifiers during heat stress, thereby modulating milk protein synthesis pathways (Böhmdorfer and Wierzbicki 2015).

#### 2.4.2 Scaffolds

LncRNAs serve as platforms to bring multiple proteins or protein complexes together, facilitating their interaction at specific genomic sites (Luo et al. 2021). A recent livestock example is lncRNA‐MEG3, which scaffolds *SUZ12* via its intrinsically disordered region to promote liquid–liquid phase separation, ultimately regulating muscle fibre composition and intramuscular fat deposition in cattle and sheep (Yao et al. 2025).

#### 2.4.3 Decoys

LncRNAs bind to proteins or other RNAs, preventing them from interacting with their natural targets (Schmitt et al. 2016). This mechanism is particularly relevant in skeletal muscle, where certain lncRNAs sequester transcription factors (TFs) that would otherwise drive premature differentiation, thereby extending the proliferative window of myoblasts and influencing final muscle mass (Cui et al. 2026).

#### 2.4.4 Guides

LncRNAs direct transcriptional complexes or chromatin modifiers to specific genomic locations (Fang and Fullwood 2016). In reproductive tissues of sheep and goats, guide lncRNAs recruit polycomb complexes to imprinted loci, contributing to the epigenetic control of ovulation rate and litter size (Lv et al. 2024).

#### 2.4.5 Architects

LncRNAs help maintain three‐dimensional genome organisation by stabilising enhancer‐promoter loops (Fang and Fullwood 2016). Although still understudied in farm animals, emerging Hi‐C data from bovine muscle cells suggest that architect lncRNAs help maintain long‐range contacts necessary for coordinated expression of myogenic gene networks (Kyung et al. 2026).

#### 2.4.6 miRNA sponges

LncRNAs act as competing endogenous RNAs (ceRNAs) by sequestering miRNAs, thereby preventing them from binding to and silencing target mRNAs (Zhang et al. 2024).

Collectively, these mechanisms are not mutually exclusive. A single lncRNA can switch between modes of action depending on developmental stage, tissue type or environmental challenge. This functional plasticity is precisely what makes lncRNAs particularly well suited to the regulation of complex, polygenic economic traits and positions them as promising targets for precision breeding. Functional studies in livestock have already provided direct evidence that lncRNAs participate in post‐transcriptional regulatory networks through interactions with miRNAs and their target genes. In sheep, the circRNA803/lncRNA MSTRG.19726 oar let‐7a CPEB1 axis has been shown to influence follicular dominance by competitively modulating CPEB1 expression (Gu et al. 2025). In goats, the lnc_2217 miR‐877‐3p YTHDF2 axis regulates zygotic genome activation, illustrating another example of lncRNA‐mediated post‐transcriptional control during early embryonic development (Chen et al. 2026). Together, these findings demonstrate that the functional outcome of an lncRNA is determined by the specific molecular partners and biological context in which it operates, reinforcing their potential as actionable targets in livestock breeding programs.

### 2.5 RNA Localisation, Secondary Structure and Stability of LncRNA

LncRNAs exhibit diverse expression patterns across tissues and subcellular compartments. This subcellular localisation and tissue specificity directly influence their regulatory impact on economically important traits in livestock. While many lncRNAs show enriched expression in the brain in model organisms, they are also widely expressed in other tissues relevant to livestock production, such as skeletal muscle, ovary, testis and mammary gland (Mercer et al. 2008; Ponting et al. 2009). For example, muscle‐enriched lncRNAs such as Lnc MyoD and MAR1 modulate myoblast differentiation and muscle fibre composition, directly affecting growth rate and carcass quality (S. Wang, Jin, et al. 2019). LncRNAs can localise to different subcellular compartments, including the nucleus, cytoplasm or specific foci, which largely determines their functional mode of action. They are often transcribed from regions near promoters and the initial exons or introns of genes (Kapranov et al. 2007). These structural elements serve as platforms for binding proteins, other RNAs or DNA, thereby mediating their regulatory activities. Stability is another key feature of lncRNAs. Unlike mRNAs, lncRNAs generally have shorter and more variable half‐lives. They can be classified into three categories based on stability: unstable (average half‐life < 2 h), stable (average half‐life > 2 h), and extremely stable (average half‐life > 16 h) (Shi et al. 2021). This variability in stability allows lncRNAs to exert rapid and context‐specific regulatory effects, which is particularly important for dynamic processes such as muscle development, follicular growth and immune responses in livestock (Shi et al. 2021). This variability enables lncRNAs to act as rapid responders to environmental stimuli (heat stress, nutrition), thereby fine‐tuning milk yield and meat quality traits in dairy and beef cattle (Shi et al. 2021).

### 2.6 Chromatin Modification by LncRNA

One of the primary mechanisms by which lncRNAs regulate gene expression is the recruitment of chromatin‐remodelling complexes (Kaneko et al. 2010). Many lncRNAs interact with PRC1 and PRC2 to mediate histone modifications, such as ubiquitination and methylation, leading to transcriptional activation or silencing (Pandey et al. 2008). A classic example is *HOTAIR*, a lncRNA transcribed from the *HOXC* locus that recruits *PRC2* to the *HOXD* locus on a different chromosome, resulting in epigenetic silencing of a large genomic region (Rinn et al. 2007; Gupta et al. 2010). Other well‐characterised lncRNAs, such as *Xist* and *H19*, play critical roles in X chromosome inactivation and genomic imprinting, respectively. These examples illustrate how lncRNAs can establish and maintain large‐scale epigenetic states through cis‐ and trans‐acting mechanisms (Chillón and Marcia 2020). In livestock species, similar chromatin‐modifying mechanisms are increasingly recognised as important regulators of muscle development, reproductive traits, and metabolic pathways, highlighting the potential of lncRNAs as epigenetic modulators in genetic improvement (see species‐specific evidence summarised in Table 2)

### 2.7 Transcriptional, Post‐Transcriptional and Post‐Translational Regulation

LncRNAs regulate transcription through multiple mechanisms, including chromatin remodelling and interactions with TFs. Some lncRNAs act by competing for TFs or recruiting them as co‐activators to enhance or repress the expression of target genes (Bhat et al. 2016; Tsai et al. 2010). At the post‐transcriptional level, lncRNAs play crucial roles in mRNA splicing, editing, transport, translation and degradation (Dykes and Emanueli 2017). Several nuclear lncRNAs, such as *NEAT1*, *MALAT1* and *MIAT*, have been shown to modulate splicing and nuclear retention of mRNAs. Although less commonly studied, some lncRNAs also influence post‐translational regulation by modulating protein stability, localisation or interactions (Tsai et al. 2010). Collectively, these multi‐level regulatory functions allow lncRNAs to exert precise and dynamic control over gene expression, which is particularly relevant for complex traits in livestock such as muscle growth, fertility and immune responses.

### 2.8 LncRNA and Regulate Cell Cycle and Apoptosis

LncRNAs play important roles in the regulation of the cell cycle and apoptosis, two processes that are critical for tissue development, homeostasis and stress responses in livestock (Jiang et al. 2021). LncRNAs can modulate apoptosis through multiple mechanisms. They may interact directly or indirectly with death receptors and members of the Bcl‐2 family, or function as ceRNAs to sponge miRNAs, thereby altering the expression of key pro‐ or anti‐apoptotic genes (Tao et al. 2019; Jiang et al. 2021). In skeletal muscle of beef cattle, lncRNAs such as *MALAT1* and *H19* modulate myoblast apoptosis to prevent excessive cell death during postnatal growth, thereby increasing muscle fibre number and improving meat yield (Jilo et al. 2026). For example, several lncRNAs have been shown to influence the survival and proliferation of muscle cells and immune cells under stress conditions. Such regulatory functions are particularly relevant for livestock traits, including muscle growth, tissue repair and disease resistance, where balanced apoptosis is essential for maintaining optimal production performance (Pal et al. 2020). Overall, by fine‐tuning cell cycle progression and apoptotic pathways, lncRNAs contribute to the adaptive responses of animals to environmental challenges, infections and metabolic demands.

### 2.9 LncRNA and Regulate Protein Translation

The genomic position of an lncRNA relative to its neighbouring genes is a key determinant of its regulatory function. Owing to the evolutionary conservation of widespread lncRNA transcription, the non‐random genomic distribution of lncRNAs is thought to represent an adaptive strategy that enables context‐specific gene regulation (Statello et al. 2021). Divergently transcribed lncRNAs, in particular, play important roles in cis‐regulation of nearby genes. This regulation can occur through two main mechanisms: (1) the lncRNA transcript itself directly modulates the expression of adjacent loci, or (2) the act of lncRNA transcription or splicing alters local chromatin structure or creates steric hindrance that influences neighbouring gene expression (Statello et al. 2021). In pig skeletal muscle, cis‐acting lncRNAs directly regulate neighbouring myogenic genes (*MyoD, Mef2*) to coordinate postnatal growth and intramuscular fat deposition (Song et al. 2024). Such cis‐regulatory mechanisms are increasingly recognised in livestock as important contributors to the fine‐tuned control of protein synthesis during muscle development, follicular maturation, and metabolic adaptation (Dykes and Emanueli 2017). More broadly, lncRNAs modulate translation initiation and elongation factors (e.g., *eIF4E*, *eIF4G*, *4E*‐*BP1*) as an additional layer of post‐transcriptional control (Karakas and Ozpolat 2021).

### 2.10 LncRNA and Gene Silencing

One of the best‐known mechanisms of lncRNA function is gene silencing. The most extensively studied example is *Xist*‐mediated X‐chromosome inactivation in female mammals. During embryonic development, *Xist* RNA spreads across one X chromosome, leading to its transcriptional silencing (Gil and Ulitsky 2020; Wutz 2011). This process also contributes to the establishment of epigenetic memory that can be maintained even after *Xist* expression decreases (Colognori et al. 2019). LncRNAs can induce gene silencing through both cis‐ and trans‐acting mechanisms. For instance, some lncRNAs form R‐loops or recruit polycomb repressive complexes (PRCs) to target promoters, resulting in histone modifications, reduced chromatin accessibility and transcriptional repression (Beckedorff et al. 2013; Latos et al. 2012). Active enhancers can also be transcribed into enhancer RNAs (eRNAs) or enhancer‐associated lncRNAs. While eRNAs are typically short and unstable, longer enhancer‐associated lncRNAs are often linked to enhancer activity and the expression of neighbouring genes (Arnold 2020; Tan and Marques 2022). In livestock, such lncRNA mediated gene silencing mechanisms are believed to play significant roles in genomic imprinting, tissue‐specific gene regulation and the control of economically important traits including muscle hypertrophy, reproductive development and metabolic adaptation. For example, imprinted lncRNAs in sheep and goats regulate ovulation rate through epigenetic silencing of reproductive genes, directly influencing litter size.

## 3 Implications of LncRNA

### 3.1 LncRNA and Complex Regulation of X Chromosome

LncRNAs exhibit diverse mechanisms of action that depend on their cellular localisation and molecular interactions. In some cases, a single lncRNA can perform multiple functions across different cellular compartments (Iyer et al. 2015). X chromosome inactivation (*XCI*) in mammals represents a classic model of lncRNA‐mediated epigenetic silencing. The lncRNA *Xist* plays a central role in this process by coating one X chromosome and recruiting chromatin‐modifying complexes to achieve gene silencing (Iyer et al. 2015; Gil and Ulitsky 2020). This mechanism has provided valuable insights into how lncRNAs establish and maintain large‐scale epigenetic states. Although *XCI* is a fundamental process in mammals, its detailed mechanisms are less directly relevant to livestock production traits (Kopp and Mendell 2018). In cattle, sheep and goats, the principles of lncRNA‐mediated epigenetic regulation extend directly to imprinted loci controlling growth and reproduction. A well‐characterised example is the ovine *DLK1‐DIO3* imprinted domain underlying the Callipyge muscle‐hypertrophy phenotype: a single cis‐acting mutation upregulates the paternally expressed *DLK1* and *RTL1* transcripts together with the maternally expressed lncRNAs *MEG3* and *MEG8*, producing a polar‐overdominant pattern of postnatal muscle hypertrophy (Charlier et al. 2001; Freking et al. 2002). A parallel imprinting‐based mechanism operates at the bovine *H19/IGF2* locus, where methylation of the H19 differentially methylated region governs the reciprocal, parent‐of‐origin‐specific expression of the *H19* lncRNA and the growth‐promoting *IGF2* gene, and altered *H19* methylation is associated with abnormal foetal growth in cattle generated by somatic cell nuclear transfer (Curchoe et al. 2009). This *H19/IGF2* imprinting mechanism itself is deeply conserved across therian mammals, having been traced back through comparative genomic analysis of marsupial and eutherian lineages (Smits et al. 2008), underscoring its fundamental role in mammalian growth regulation, including in livestock species.

### 3.2 LncRNA in Epigenetics

LncRNAs play a central role in epigenetic regulation, and recent research has begun to reveal a more unified picture of how they operate. The first clear connections between lncRNAs and major epigenetic silencing processes in mammals genomic imprinting and X chromosome inactivation came from the discovery of *H19* and *Xist*, respectively. The *H19* lncRNA, which also hosts the imprinted miRNA *miR‐675*, is thought to be involved in growth regulation. However, its functions in the cytoplasm are still not fully understood, particularly how it escapes Nonsense‐Mediated Decay (NMD) after being spliced and exported from the nucleus (Ponting et al. 2009). Studies in embryos have shown that components of the nuclear mRNA degradation and NMD pathways help control *H19* levels during embryonic stem cell differentiation (Ciaudo et al. 2006). While it shares some similarities with *H19*, such as being spliced, polyadenylated and regulated by NMD, *Xist* remains in the nucleus and coats the X chromosome to initiate chromosome‐wide silencing (Brown and Willard 1994; Clemson et al. 1996). A critical region of *Xist* recruits components of the Polycomb Repressive Complex 2 (PRC2), promoting *H3K27me3* deposition and the establishment of a repressive chromatin state. Subsequently, DNA methylation and other histone modifications contribute to the maintenance of the inactive X chromosome and the stability of the epigenetic state through cell division (Wutz and Gribnau 2007; Zhao et al. 2008). Interestingly, deletion of *Xist* in differentiated somatic cells does not necessarily lead to immediate reactivation of the inactive X chromosome, indicating that additional epigenetic mechanisms can contribute to maintenance of the silenced state (Brown and Willard 1994; Wang et al. 2021). Similar principles apply to autosomal genomic imprinting. For example, transcription across the Airn locus contributes to paternal silencing of *Igf2r*, illustrating how lncRNA transcription can establish allele‐specific gene regulation (Pauler et al. 2007). In germ cells, lncRNAs associated with imprinting control regions can also contribute to the establishment of DNA methylation patterns (Chotalia et al. 2009). Overall, these findings demonstrate that lncRNAs can participate in the establishment and maintenance of epigenetic states through interactions with chromatin‐modifying complexes, DNA methylation machinery and transcriptional regulatory processes. Importantly, these principles have also been investigated in domestic animals. In cattle, *Xist* expression has been detected during preimplantation embryo development, and its temporal pattern is consistent with a role in the initiation of X chromosome inactivation and dosage compensation (De La Fuente et al. 1999). In addition, genomic imprinting involving the *H19‐IGF2* and *IGF2R* loci has been demonstrated in sheep, indicating that epigenetic mechanisms involving imprinted loci are evolutionarily conserved in livestock. Aberrant DNA methylation at the *IGF2R* differentially methylated region has also been observed in cloned sheep, highlighting the sensitivity of these epigenetic regulatory regions to developmental manipulation (Young et al. 2003). Beyond classical imprinting and X chromosome inactivation, functional studies in livestock have demonstrated that lncRNAs are associated with economically important biological processes. For example, *H19* is highly expressed in bovine skeletal muscle and promotes the differentiation of bovine skeletal muscle satellite cells, partly through suppression of the *Sirt1/FoxO1* pathway (Xu et al. 2017). Other lncRNAs have also been implicated in bovine myoblast proliferation and differentiation, supporting an important regulatory role for lncRNAs in muscle development and, consequently, growth and meat‐production traits (M. Li, Gao, et al. 2019). In goats, transcriptome studies of ovarian tissues have identified differentially expressed lncRNAs associated with follicular development and fecundity‐related phenotypes. For example, several lncRNAs were differentially expressed between high‐ and low‐fecundity goats, suggesting their potential involvement in ovarian and follicular development (Li et al. 2021a). Similarly, lncRNA profiles of pre‐ovulatory goat ovarian follicles have identified candidate lncRNAs involved in follicular regulation, including XR_311113.2, which was experimentally shown to act as a miRNA sponge (Tao et al. 2022). In bovine adipose tissue, increasing evidence indicates that lncRNAs participate in adipocyte differentiation and fat deposition through interactions with transcriptional and epigenetic regulatory pathways (Jilo et al. 2024). Recent studies have identified specific lncRNAs that regulate bovine adipogenesis and have highlighted their potential relevance to economically important traits such as fat deposition and meat quality (Jilo et al. 2024). Collectively, these findings demonstrate that the regulatory principles established through studies of lncRNA‐mediated epigenetic regulation are relevant to domestic animals and provide a molecular framework for understanding the regulation of growth, reproduction, adipogenesis and other economically important livestock traits.

### 3.3 LncRNA and Diseases

Many lncRNAs have been strongly associated with various human diseases, especially cancer (Gutschner and Diederichs 2012). Their dysregulation has been observed in glioblastoma, breast cancer, colorectal cancer, liver cancer and leukemia (Fang and Fullwood 2016). In many cases, the initial evidence came from differences in expression levels between diseased and healthy tissues (Szymanski et al. 2005). A well‐known example is the elevated antisense transcript of the *β‐secretase‐1 (BACE1)* gene, which has been linked to the progression of Alzheimer's disease (Faghihi et al. 2008). It is important to note, however, that changes in lncRNA expression do not always indicate a causal role. Recent studies show that mutations within lncRNA loci themselves can contribute to disease. Such dysregulation can promote cell proliferation, resistance to apoptosis, angiogenesis and evasion of tumour suppressor pathways (Dhanoa et al. 2018). Prominent oncogenic lncRNAs identified in human cancers include *ANRASSF1*, *ANRIL*, *HOTAIR*, *CCAT2* and *HOTAIRM1*, which have been implicated in cancer‐related processes through diverse regulatory mechanisms (Beckedorff et al. 2013; Bhan et al. 2013(. In livestock, the role of lncRNAs in disease resistance is gaining attention. A recent study on Holstein cows with different stages of paratuberculosis (Johne's disease), a costly chronic infection, used integrated RNA‐seq and small RNA‐seq on jejunal tissue. The researchers identified differentially expressed lncRNAs, miRNAs and mRNAs linked to susceptibility or resistance to Mycobacterium Avium subspecies Paratuberculosis (MAP). They constructed a ceRNA network highlighting six lncRNAs and 48 miRNAs that may regulate important immune genes such as *BOLA‐DQB* and *PPARG*. These findings offer promising new targets for breeding cattle with improved disease resistance (Gao et al. 2025).

### 3.4 LncRNAs in Autoimmune Diseases

Autoimmune diseases often result from subtle disruptions in the regulation of immune cells, particularly B and T lymphocytes. Emerging evidence indicates that lncRNAs play important roles in immune cell function and immune tolerance. Similar to microRNAs, dysregulation of certain lncRNAs has been linked to the development of autoimmune disorders in both humans and mice. Both increased and decreased expression of specific lncRNAs have been associated with various autoimmune conditions. For example, upregulation of the lncRNA *GAS5* has been observed in tuberculosis, while its downregulation has been reported in rheumatoid arthritis (Mayama et al. 2016). Although most research in this area has been conducted in humans and mouse models, these findings provide valuable insights into the potential involvement of lncRNAs in immune regulation and inflammatory responses in livestock. Given the economic impact of infectious and immune‐related diseases in farm animals (Li et al. 2025), understanding lncRNA‐mediated immune modulation could open new avenues for improving disease resistance through genetic selection and breeding programs.

### 3.5 LncRNAs in Chromatin Looping and Enhancer Function

LncRNAs can also regulate gene expression through chromatin looping and enhancer activity. eRNAs and enhancer‐associated lncRNAs participate in long‐range chromatin interactions that coordinate transcriptional activation or repression (Fang and Fullwood 2016). Although most mechanistic studies have been performed in human cancer models (e.g., *HOTTIP* in *HOXA* gene regulation) (Wang et al. 2011), these principles of chromatin architecture are conserved across mammals. A positive correlation has been reported between eRNA abundance and target gene expression, as seen in the prostate‐specific antigen (PSA) gene and other p53‐bound enhancers (Hsieh et al. 2014; Melo et al. 2013). Protein complexes such as cohesin can further stabilise these loops (Ørom and Shiekhattar 2013). In livestock, similar lncRNA‐mediated chromatin looping mechanisms are believed to play important roles in regulating complex traits such as muscle development, adipogenesis and reproductive gene networks. However, direct experimental evidence for these mechanisms in livestock remains limited and further functional studies are needed to determine their relevance to economically important traits.

### 3.6 LncRNA in Crispr

Perturbation of lncRNA expression can be achieved through several approaches, including CRISPR‐based genome editing of lncRNA loci (Ho et al. 2015). Antisense Oligonucleotides (*ASOs*) provide another strategy capable of targeting both cytoplasmic and nuclear lncRNAs. The CRISPR system, originally derived from bacterial and archaeal immune defenses that use short RNAs to degrade invading nucleic acids (Mali et al. 2013), has been successfully adapted to excise lncRNAs for functional studies. Additionally, catalytically inactive Cas9 (dCas9) fused with transcriptional activators such as *VP64* can be used to upregulate lncRNA expression (Maeder et al. 2013; Arroyo‐Olarte et al. 2021; Newsom et al. 2021). More recently, RNA targeting CRISPR systems such as Cas13 have emerged as powerful tools for directly perturbing lncRNA transcripts without altering the underlying genomic DNA. This approach significantly reduces off‐target effects on nearby coding genes and enables high‐throughput functional screening, as demonstrated by transcriptome‐scale studies in human cells (Liang et al. 2026). Adaptation of these RNA targeting technologies to livestock species is expected to greatly accelerate the discovery and functional validation of lncRNAs that influence economically important traits such as growth, reproduction, disease resistance and meat quality (Li et al. 2023).

### 3.7 LncRNA In Reproduction

#### 3.7.1 LncRNAs in Spermatogonial Stem Cells (SSC)

Spermatogenesis is a complex and continuous process in which spermatogonial stem cells (SSCs) ultimately give rise to mature spermatozoa. Now, LncRNAs are recognised as broad regulators of reproductive and developmental processes across mammals, spanning gametogenesis, fertilisation and embryogenesis (Taylor et al. 2015). LncRNAs are increasingly recognised as key components of the RNA‐protein regulatory networks that govern animal sperm development (Zhang et al. 2016). LncRNAs play important regulatory roles in SSC self‐renewal, differentiation and lineage progression. For example, *GDNF*‐responsive lncRNAs have been shown to influence SSC fate decisions (Li et al. 2016). In vitro studies in mice demonstrated that the lncRNA AK015322 promotes proliferation of the SSC‐derived C18‐4 cell line, suggesting its involvement in maintaining SSC populations (Hu et al. 2017). However, no ortholog or functional study of AK015322 has been reported in livestock or poultry species to date, and its direct relevance to economically important traits remains unknown. In contrast, several lncRNAs have been associated with testicular development, spermatogenesis and male reproductive function in livestock. In cattle, comparative transcriptomic analysis of immature and sexually mature testes identified numerous differentially expressed lncRNAs, including transcripts associated with *COX7A2, TRIM37* and *PRM2*, which were associated with testicular development, sexual maturation and spermatogenesis (X. Wang, Yang, Guo, et al. 2019). In sheep, transcriptomic analyses have also revealed extensive changes in testicular lncRNA expression during sexual maturation, with lncRNAs associated with pathways involved in cell cycle regulation, metabolism and spermatogenesis (Li et al. 2026). In chickens, comparative lncRNA and mRNA profiling of testes with divergent sperm motility identified candidate transcripts linked to ATP binding and cilium assembly, pointing to lncRNA involvement in sperm functional quality (Liu et al. 2017). These findings suggest that lncRNAs may contribute to the regulation of testicular development and male reproductive capacity in livestock, although functional validation remains limited for many candidates. Beyond testicular development, lncRNAs are also involved in early embryonic development in domestic animals. A recent study identified the novel lncRNA lnc_2217 as an important regulator of Zygotic Genome Activation (ZGA) in goats. Knockdown of lnc_2217 by siRNA microinjection significantly reduced the blastocyst formation rate from approximately 32.5% to 13.9%. This developmental impairment was associated with reduced global de novo RNA synthesis and excessive degradation of maternal mRNAs at the 8‐cell stage. Mechanistically, lnc_2217 functions as a ceRNA that sequesters miR‐877‐3p and thereby sustains the expression of the m6A reader *YTHDF2*. This regulatory axis contributes to the appropriate clearance of maternal transcripts, including *ZAR1, YAP1* and *UHRF1*, during the maternal‐to‐zygotic transition (MZT) and supports successful embryonic development (Chen et al. 2026). Collectively, these findings provide direct evidence that lncRNAs participate in important reproductive processes in domestic animals, including testicular development, spermatogenesis and early embryonic development.

#### 3.7.2 LncRNAs in Fertility and Germ Cell Specification

LncRNAs have long been recognised for their roles in genomic imprinting and X chromosome inactivation. Recent studies show that they operate through multiple mechanisms, including acting as competitors or recruiters of epigenetic modifiers and serving as precursors for small ncRNAs. LncRNAs are also emerging as important regulators of Primordial Germ Cell (PGC) specification and later stages of gametogenesis. Whole‐transcriptome analyses in livestock species have substantially advanced our understanding of lncRNA functions in reproduction. In sheep, comparative ovarian transcriptomics between high‐fecundity (Small Tail Han) and low‐fecundity (Ujumqin) breeds revealed a comprehensive ceRNA network. A key validated axis, *circRNA803/lncRNA MSTRG.19726–oar‐let‐7a CPEB1*, was shown to regulate follicular dominance, a critical factor influencing ovulation rate and litter size, through competitive sponging of oar‐let‐7a to modulate *CPEB1* expression (Gu et al. 2025). Similarly, in chickens, whole‐transcriptome sequencing across the Hypothalamus Pituitary Ovarian (HPO) axis identified numerous differentially expressed lncRNAs, miRNAs and mRNAs associated with gonadal differentiation and sexually dimorphic patterns (Zou et al. 2020). Comparable whole‐transcriptome analyses of atrophic ovaries in broody hens have further implicated lncRNAs in regulatory pathways controlling granulosa cell proliferation and apoptosis, directly affecting laying performance (Liu et al. 2018).

Key regulatory networks targeting genes such as *GATA4*, *SMAD3* and *FOXL2* have been uncovered, providing promising molecular targets for improving reproductive efficiency through marker‐assisted selection (P. Li, Chu, et al. 2025).

### 3.8 LncRNA and Skeletal Muscle

Skeletal muscle is one of the most economically important tissues in livestock, as it directly determines meat yield, carcass composition and overall product quality. Therefore, understanding lncRNA‐mediated regulation of muscle development has become a major priority in animal breeding research (Cui et al. 2026). Over the past decade, thousands of lncRNAs have been identified in skeletal muscle across species. Although the biological functions of most remain poorly characterised, current evidence shows that lncRNAs regulate muscle development through diverse mechanisms, including chromatin remodeling, transcriptional activation, acting as molecular sponges, competitive protein binding, modulation of mRNA translation and control of protein stability (Neguembor et al. 2014; Zhang et al. 2018). For example, the lncRNA Chronos increases with age in mouse skeletal muscle and suppresses hypertrophy by repressing *Bmp7* expression, while the muscle‐enriched lncRNA *MAR1* promotes differentiation and regeneration via regulation of Wnt5a (Neppl et al. 2017). Several lncRNAs are also involved in postnatal muscle growth. Notable examples include *MALAT1, NEAT1* (isoforms v1 and v2), LncMyoD and Linc‐MD1, which show dynamic expression during early postnatal development in sheep, particularly at the Callipyge locus (Mikovic et al. 2018). In particular, LncMyoD, directly activated by the myogenic factor *MyoD*, plays a key role in promoting cell‐cycle exit and myogenic differentiation (Gong et al. 2015). The imprinted lncRNA *H19* regulates myoblast differentiation through multiple pathways, including recruitment of PRC2, sponging of let‐7 miRNA and modulation of mRNA stability via KSRP (Kallen et al. 2013).

In livestock species, transcriptome studies have provided valuable insights into lncRNA functions in muscle biology. In sheep, RNA‐seq identified 1,566 lncRNAs, with 404 differentially expressed between prenatal and postnatal skeletal muscle. Many of these lncRNAs showed potential cis‐regulatory interactions with key myogenic genes such as *Mef2* and *MyoD* (C.‐Y. Li, Li, Liu, et al. 2019). In chickens, comparative analyses revealed conserved synteny and co‐expression patterns between lncRNAs and neighbouring mRNAs, supporting their involvement in muscle development and differentiation (Muret et al. 2017; Li et al. 2017). Beyond classical mechanisms, a novel layer of regulation has recently emerged through Liquid‐Liquid Phase Separation (LLPS). The lncRNA *MEG3* regulates skeletal muscle mass and metabolic homeostasis by modulating LLPS of the *SUZ12* protein via its intrinsically disordered region (IDR). This process influences slow‐twitch fibre proportion, mitochondrial function and intramuscular fat accumulation, offering new insights into meat quality traits (Yao et al. 2025).

Recent whole‐transcriptome studies in beef cattle have further revealed complex regulatory networks between muscle and adipose tissues. In Qinchuan cattle, hundreds of differentially expressed lncRNAs, circRNAs and miRNAs were identified, with ceRNA networks highlighting key axes such as MSTRG.28538.1/pma‐miR‐145‐5p/EYA4 that may coordinate muscle growth and fat deposition (Qiu et al. 2025). In a refined bovine skeletal muscle cell and intramuscular adipocyte co‐culture model, critical lncRNAs involved in myogenesis‐adipogenesis crosstalk were screened, providing valuable resources for understanding intramuscular fat (marbling) development without compromising muscle growth, a key target for genetic improvement in beef cattle (Yang et al. 2025).

## 4 LncRNA Databases

In addition to high‐throughput experimental methods, numerous specialised databases and computational resources now support lncRNA research in livestock. Historically, lncRNAdb served as the first major repository of manually curated functional lncRNAs (Quek et al. 2015). More recent platforms, such as LNCipedia v5.2, provide expanded annotation with over 127,000 human transcripts and detailed information on sequence features, conservation and coding potential (Volders et al. 2019). For transcriptional regulation, ChIPBase v3.0 integrates large‐scale ChIP‐seq data to link lncRNAs, miRNAs and TFs to specific genomic loci and co‐expression networks (Huang et al. 2023). NONCODE v7.0 stands out for its comprehensive coverage of non‐coding RNAs across species, incorporating single‐cell RNA‐seq data and extending annotation to livestock species (Liu et al. 2026). The Rfam database (version 15.1, January 2026) offers curated multiple sequence alignments and secondary structure models for 4227 RNA families (Ontiveros‐Palacios et al. 2025). Experimentally validated resources include EVLncRNAs 3.0, which focuses on functional lncRNAs validated by low‐throughput experiments (Zhou et al. 2024) and LncRNADisease v3.0, which curates disease‐associated lncRNAs and circRNAs (Lin et al. 2024). GO annotations further help interpret the biological roles of lncRNAs by linking them to cellular components, biological processes and molecular functions (Chodurska and Kunej 2025). In parallel, general genomic platforms such as Ensembl Release 116 (June 2026) (Harrison et al. 2024), NCBI RefSeq/GenBank (Sayers et al. 2025), Gene Cards (Stelzer et al. 2016) and the *HGNC* (Seal et al. 2023) provide complementary genome annotation. These resources together form a powerful ecosystem for lncRNA discovery, annotation, functional validation and comparative studies in livestock and other species (Table 1).

**TABLE 1:** Major lncRNA databases and their relevance to livestock genomics.

Database	Latest version / Year	Type of data	Key features	Livestock relevance	URL
NONCODE	v7.0 (2026)	RNA‐seq (bulk and single‐cell) + expression profiles	Comprehensive annotation, co‐expression networks, immune and developmental data	High—Covers cattle, pig, sheep, chicken	http://www.noncode.org
LNCipedia	v5.2 (2026)	Sequence and structural annotation	127,802 transcripts, coding potential, detailed annotations	Moderate—Primarily human, useful for comparative studies	https://lncipedia.org
RNAcentral	Release 27 (2026)	Integrated ncRNA sequences from >50 specialist databases	Unified platform with standardised identifiers	High—Multi‐species including livestock	https://rnacentral.org
EVLncRNAs	v3.0 (2024)	Experimentally validated functional lncRNAs	Low‐throughput validation, interactions and structures	Moderate—Includes multi‐species validation	http://biophy.dzu.edu.cn/EVLncRNAs
LncRNADisease	v3.0 (2024)	Disease association data	25,440 curated entries with experimentally supported associations	Indirect—Useful for comparative pathology	http://www.cuilab.cn/lncrnadisease
ChIPBase	v3.0 (2023)	ChIP‐seq, RNA‐seq & regulatory data	TF‐lncRNA networks, enhancer annotations and co‐expression networks	High—Supports cross‐species inference	http://rna.sysu.edu.cn/chipbase
Ensembl	Release 116 (June 2026)	Genome annotation, comparative genomics, variants	Genomic coordinates, conservation, variant information	Very High—Excellent livestock assemblies	https://www.ensembl.org
NCBI (RefSeq/GenBank)	Continuous (RefSeq v235, 2026)	Sequence records and literature	Integrated genomic and literature resources	Very High—Comprehensive livestock genomes	https://www.ncbi.nlm.nih.gov
GeneCards	v5.26 (2026)	Gene‐centric functional summaries	Expression, pathways, disease links and functional information	Moderate—Useful as comparative reference	https://www.genecards.org
HGNC	Continuous update (2026)	Gene nomenclature and annotation	Approved gene symbols, locus information and ortholog mapping	Limited—Helpful for standardised nomenclature	https://www.genenames.org

## 5 CircRNAs: A Complementary Class of NcRNAs

In addition to lncRNAs, circRNAs have gained considerable attention in recent years as another important class of regulatory ncRNAs (Figure 2). Unlike linear lncRNAs, circRNAs possess a covalently closed loop structure generated by back‐splicing. This unique circular configuration makes them highly resistant to exonuclease degradation, resulting in greater stability and making them promising candidates as molecular biomarkers (Chen 2020; Kirgiafini et al. 2024). The most well‐characterised function of circRNAs is acting as miRNA in ceRNAs (Kirgiafini et al. 2024). This mechanism is functionally similar to that of many lncRNAs, and both are frequently integrated into shared ceRNA networks that fine‐tune gene expression (Statello et al. 2021). By sequestering miRNAs, circRNAs indirectly regulate the expression of target genes involved in key livestock production traits, including skeletal muscle development, adipogenesis and reproduction (Yang et al. 2021). Based on their biogenesis and structure, circRNAs are generally classified into three major types: exonic circRNAs (EcircRNAs), circular intronic RNAs (ciRNAs) and exon–intron circRNAs (EIciRNAs) (Figure 2). EcircRNAs are mostly cytoplasmic and often function as miRNA sponges, while some also have protein‐coding potential. ciRNAs and EIciRNAs are primarily nuclear and can regulate transcription of their host genes. Representative examples include circHIPK3 (EcircRNA), ciANKRD52 (ciRNA) and *circEIF3J (EIciRNA)* (Li and Wang 2024). In livestock species, a growing number of circRNAs have been linked to economically important traits (Sun et al. 2023). Genome‐wide profiling in cattle has identified numerous differentially expressed circRNAs during skeletal muscle development, adipogenesis, and mammary gland function, highlighting their roles in growth performance, meat quality and lactation (Gao et al. 2022; Yan et al. 2023; X. Li, Chen, et al. 2025). In sheep, comprehensive multi‐tissue analyses have identified thousands of circRNAs with strong tissue‐specific expression patterns. These circRNAs are associated with muscle growth, reproductive physiology and immune responses (Bakhtiarizade et al. 2024; Varela‐Martínez et al. 2021). In pigs, circRNAs have been implicated in adipose tissue deposition, skeletal muscle growth and reproductive performance (Li et al. 2022; Ma et al. 2022). Similarly, studies in poultry have revealed circRNAs involved in egg‐laying performance, stress response and immune regulation (You et al. 2025). Importantly, circRNAs are often studied together with lncRNAs to construct integrated regulatory networks, providing deeper insights into the molecular control of complex livestock traits (Yang et al. 2022). Despite these advances, functional characterisation of circRNAs in livestock remains limited, and most studies are still descriptive. Future research using high‐throughput approaches, such as CRISPR‐Cas13‐based RNA targeting and systematic loss‐of‐function screens, will be essential to uncover their precise biological roles. Integrating circRNA and lncRNA analyses will provide a more comprehensive understanding of the ncRNA landscape and its potential for precision breeding and genetic improvement in livestock.

## 6 Research Advances of LncRNAs in Animal Science

Since the early 2010s, rapid progress in high‐throughput sequencing technologies has dramatically expanded our knowledge of lncRNAs in livestock and other animal species (Figure 3). Genome‐wide transcriptomic studies have revealed extensive transcription in non‐coding regions across farm animal genomes, leading to the identification of thousands of lncRNA transcripts with potential regulatory functions. Early large‐scale efforts reported over 53,000 lncRNA records in pigs, approximately 12,850 in chickens and 22,227 in cattle (NONCODE v5.0) (Lagarrigue et al. 2021). These discoveries highlighted the widespread presence of lncRNAs and their potential importance in regulating economically significant traits. Across livestock species, a consistent pattern emerges: lncRNAs primarily regulate economic traits through three recurring mechanistic themes (i) ceRNA networks that fine‐tune miRNA activity during muscle and follicular development, (ii) chromatin remodelling that controls myogenic and adipogenic gene programs and (iii) epigenetic scaffolding that stabilises tissue‐specific expression states. For example, in cattle, the lncRNA *MEG3* regulates muscle mass and intramuscular fat by facilitating *SUZ12* liquid–liquid phase separation (Yao et al. 2025), while in sheep, the circRNA803/lncRNA MSTRG.19726‐oar‐let‐7a‐CPEB1 axis controls follicular dominance and litter size (Gu et al. 2025). In pigs, cis‐acting lncRNAs regulate neighbouring myogenic genes such as MyoD and Mef2 to coordinate postnatal growth and intramuscular fat deposition. In chickens, key lncRNA‐miRNA‐mRNA networks targeting *GATA4, SMAD3* and *FOXL2* influence laying performance. These conserved mechanistic themes, rather than species‐specific lists of identified transcripts, provide a clearer framework for prioritising candidates for functional validation and genomic selection. Accumulating evidence shows that lncRNAs participate in a wide range of biological processes critical for animal production, including growth and muscle development, reproductive performance, lipid metabolism, immune regulation and milk production. The major functional categories and representative mechanisms of lncRNAs in these traits are summarised in Table 2. As shown in the table, lncRNAs influence livestock physiology through diverse mechanisms such as chromatin remodelling, transcriptional regulation and ceRNA interactions with miRNAs.

**FIGURE 3:**
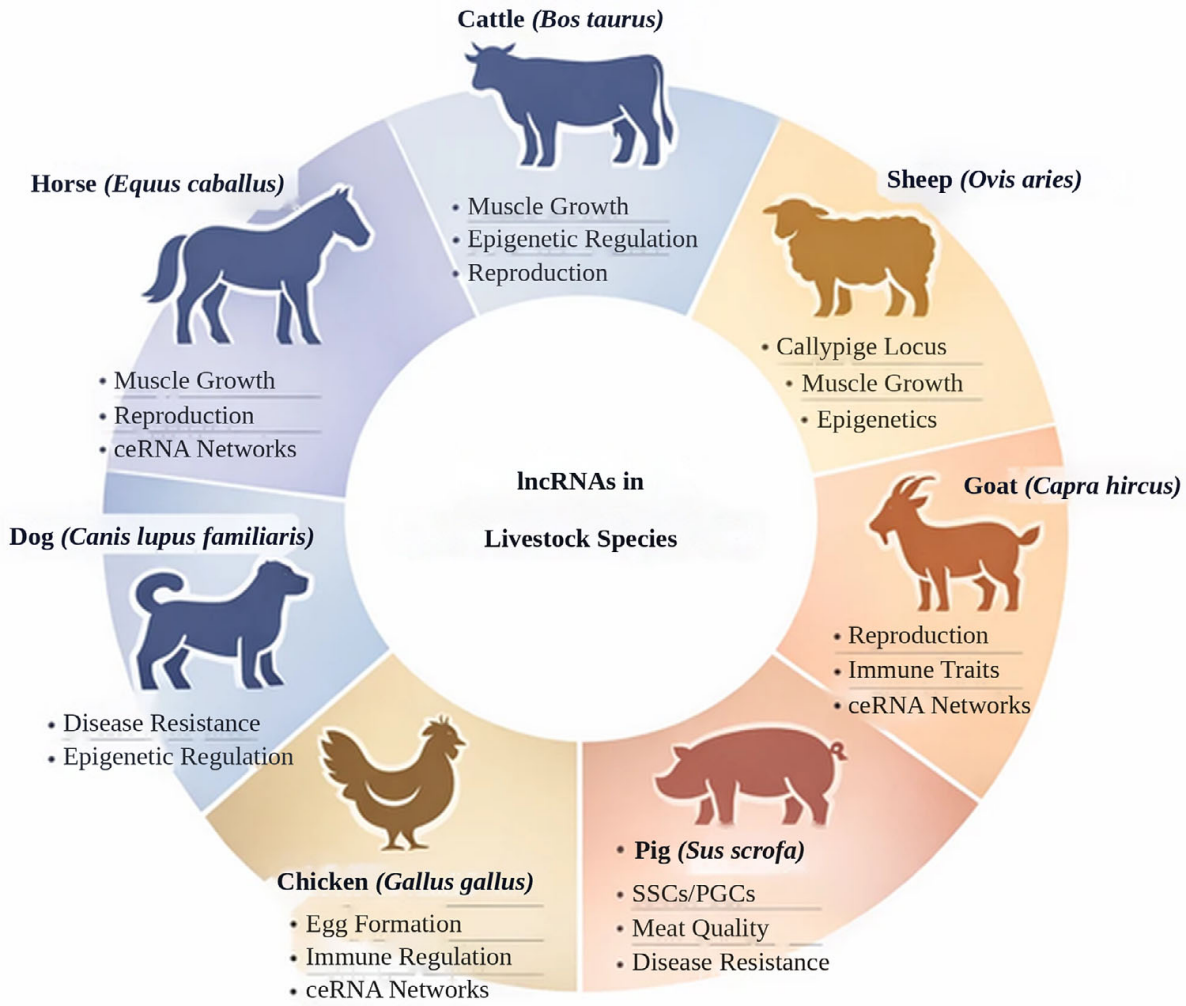
Overview of lncRNA‐associated biological functions across major livestock species. The figure summarises the key physiological and trait‐related processes in which lncRNAs have been functionally implicated in five livestock species: cattle (*Bos taurus*), sheep (*Ovis aries*), goat (*Capra hircus*), chicken (*Gallus gallus*) and pig (*Sus scrofa*). In cattle, lncRNAs are associated with muscle growth, epigenetic regulation and reproduction. In sheep, they are linked to the Callipyge locus, muscle growth and epigenetic mechanisms. In goat, lncRNAs play roles in reproduction, immune traits and ceRNA networks. In chicken, lncRNAs contribute to egg formation, immune regulation and ceRNA networks. In pig, they are associated with spermatogonial stem cells (SSCs)/Primordial Germ Cells (PGCs), meat quality and disease resistance. This species‐specific distribution highlights both conserved and divergent roles of lncRNAs in economically important traits and provides a roadmap for functional investigations in livestock genomics.

**TABLE 2:** Major biological functions of lncRNAs involved in economically important traits in livestock species

Biological function	Mechanistic roles of lncRNAs	Example traits in livestock	Representative studies
Growth and muscle development	Regulation of myogenesis; ceRNA activity; chromatin remodelling; LLPS	Growth rate, carcass traits, muscle fibre formation, IMF	Li et al. 2020 (cattle); Sun et al. 2017 (pig); D. Li, Li, Jiang, et al. (2019) (chicken); Yao et al. 2025 (MEG3–SUZ12)
Reproductive performance	Regulation of folliculogenesis and spermatogenesis; ceRNA networks; HPG/HPO axis signalling	Fecundity, litter size, puberty onset, sperm quality	Miao et al. 2016 (sheep); Gu et al. 2025 (sheep); Chen et al. 2026 (goat); X. Wang, Yang, Guo, et al. (2019) (cattle)
Lipid metabolism and adipogenesis	Regulation of adipocyte differentiation; ceRNA control of lipogenic genes	Backfat thickness, intramuscular fat, body composition	Kumar et al. 2019 (pig); Muret et al. 2017 (chicken); Miao et al. 2018 (pig)
Immune function and disease resistance	Modulation of innate/adaptive immunity; cytokine regulation; antiviral responses	Resistance to Marek's disease, IBV, Johne's disease, mastitis	Zhang et al. 2021 (chicken); Li et al. 2021b (chicken); Gao et al. 2025 (cattle); Özdemir and Altun 2020 (cattle)
Milk production and mammary gland biology	Regulation of mammary epithelial proliferation and milk protein/lipid synthesis	Milk yield, milk components, mammary development	Tong et al. 2017; Zeng et al. 2019 (cattle)
Energy metabolism and nutrient utilisation	Regulation of metabolic pathways and feed‐induced tissue remodelling	Feed efficiency, rumen/ileum development	Ibeagha‐Awemu et al. (2018) (cattle); Weikard et al. (2018) (cattle)
Tissue development and organogenesis	Regulation of cell differentiation and organ‐specific signalling	Skin pigmentation, embryonic development, rumen development	Ren et al. 2016 (goat); Weikard et al. 2018 (cattle)

In cattle, one of the first genome‐wide studies identified 449 lncRNAs in intergenic regions (Huang et al. 2012), laying the foundation for subsequent research. Later studies linked lncRNAs to growth performance, meat quality, testicular development, skin pigmentation, rumen and ileum function, and mammary gland development and lactation, acting mainly through ceRNA‐mediated miRNA sponging and chromatin remodelling that modulate myogenic and lipogenic gene networks (Li et al. 2020; Choi et al. 2019; X. Wang, Yang, Guo, et al. 2019; Weikard et al. 2018; Tong et al. 2017; Zeng et al. 2019). In pigs, numerous lncRNAs have been associated with growth rate, intramuscular fat deposition, backfat thickness and reproductive processes such as ovarian development and endometrial regulation during pregnancy, with several backfat and intramuscular‐fat‐associated lncRNAs functioning as ceRNAs that sequester adipogenesis‐related miRNAs (Sun et al. 2017; Zou et al. 2017; Miao et al. 2018; Kumar et al. 2019; Wang et al. 2016; Z. Wang, Yang, Li, et al. 2019).

In poultry, particularly chickens, lncRNA research has focused on meat quality, egg production, gonadal development and immune responses to diseases such as Marek's disease and infectious bronchitis virus, with egg‐production‐associated lncRNAs frequently acting on HPG/HPO‐axis genes (e.g., *GATA4, SMAD3, FOXL2*) via ceRNA sponging (D. Li, Li, Jiang, et al. 2019; Muret et al. 2017; Zhang et al. 2021; Li et al. 2021b). In small ruminants like sheep and goats, lncRNAs have been implicated in fecundity, testicular development, puberty onset and skin pigmentation. ceRNA networks involving lncRNAs and miRNAs appear to play significant roles in reproductive performance (Miao et al. 2016; Gao et al. 2017; Ren et al. 2016; Zhang et al. 2017). In horses, large‐scale transcriptomic studies have identified more than 20,000 candidate lncRNAs, establishing a foundational resource for understanding gene regulation and trait‐associated molecular mechanisms in equine species (Scott et al. 2017).

While many foundational insights originated from mouse models and human studies (Guttman et al. 2010; Sigova et al. 2013), comparative transcriptomic analyses in livestock are increasingly revealing species‐specific and economically relevant roles.

As illustrated in Figure 3, the ceRNA and scaffold modes are the most frequently documented mechanisms in livestock traits, particularly in relation to muscle hypertrophy and fecundity. These conserved regulatory themes provide a useful framework for understanding how lncRNAs exert their effects across different biological contexts and species.

Collectively, Table 2 reveals two recurring patterns across livestock species. First, ceRNA (miRNA‐sponge) activity is the most broadly reported mechanism, recurring across growth/muscle, reproductive, lipid‐metabolism and immune‐related lncRNAs. This suggests that ceRNA activity may serve as a generalisable entry point for functional screening across traits.

Second, the specific molecular partners differ substantially by trait and species: muscle‐related lncRNAs converge on core myogenic regulators (*MyoD*, *Mef2*), reproductive lncRNAs on HPG‐axis and follicular genes (*GATA4*, *SMAD3*, *FOXL2* and *CPEB1*), and lipid‐metabolism lncRNAs on adipogenic TFs. This pattern indicates that while the regulatory logic of lncRNAs is broadly conserved, their functional targets are trait‐ and tissue‐specific. Consequently, a prioritise‐validate‐integrate framework is more appropriate than a one‐size‐fits‐all annotation strategy. Overall, lncRNAs represent a crucial regulatory layer influencing animal growth, reproduction, metabolism, immunity and disease resistance. These advances open the way for incorporating lncRNA‐based information into future genetic improvement programs, although substantial functional validation is still required.

## 7 Challenges and Future Recommendations

Despite substantial progress in identifying lncRNAs and exploring their regulatory potential, much of their biology in livestock species remains poorly understood. High‐throughput sequencing has led to the discovery of thousands of lncRNAs in cattle, sheep, goats, pigs, poultry and other farm animals (Figure 4). However, the majority of these transcripts still lack experimental functional validation. Most current knowledge is based on expression profiling and bioinformatic predictions, leaving clear causal relationships between specific lncRNAs and economically important traits as a major challenge. Another key difficulty lies in the remarkable diversity of lncRNA mechanisms. Depending on cellular context, they can regulate gene expression through chromatin remodeling, transcriptional control, molecular scaffolding, ceRNA activity, protein interactions and even LLPS. This functional versatility makes systematic characterisation particularly demanding. Characterising lncRNA interaction networks is a high research priority. Techniques such as chromatin isolation by RNA purification (ChIRP) and similar methods offer powerful tools to identify lncRNA‐associated chromatin regions and protein partners. Wider application of these approaches in livestock models will significantly advance our understanding of their regulatory networks. Functional validation remains a critical bottleneck. While CRISPR/Cas9 has revolutionised genomics, its use for lncRNAs is complicated because many lncRNA loci overlap with important regulatory DNA elements. RNA‐targeting systems such as CRISPR‐Cas13 provide a promising alternative by enabling specific transcript‐level perturbation without altering the genomic DNA. Adapting these tools to livestock species is expected to greatly accelerate functional studies. Future progress will depend on integrating multiple layers of biological data: transcriptomics, epigenomics, chromatin accessibility, proteomics, metabolomics and genome‐wide association studies (GWAS). Artificial Intelligence (AI) and machine learning (ML) are also expected to play an increasingly important role by improving predictions of lncRNA structure, function and regulatory potential, helping researchers prioritise the most promising candidates for validation. Ultimately, translating these fundamental discoveries into practical applications is a central goal of livestock genomics. A deeper understanding of lncRNA regulatory networks could lead to the identification of novel biomarkers and functional variants associated with growth rate, meat quality, reproductive efficiency, disease resistance, feed utilisation and environmental adaptation. Incorporating lncRNA information into genomic selection programs has the potential to improve breeding value prediction and support more efficient, sustainable livestock breeding strategies. In summary, lncRNAs have emerged as a vital regulatory layer in livestock genomes. Continued advances in functional genomics, genome editing, multi‐omics integration and computational biology will be essential to fully unlock their potential for genetic improvement and precision breeding in the coming years. Moreover, the limited availability of high‐quality genome assemblies and functional annotation tools for many livestock breeds remains a bottleneck. International collaborative efforts, such as the Functional Annotation of Animal Genomes (FAANG) project, are expected to significantly improve this situation.

**FIGURE 4:**
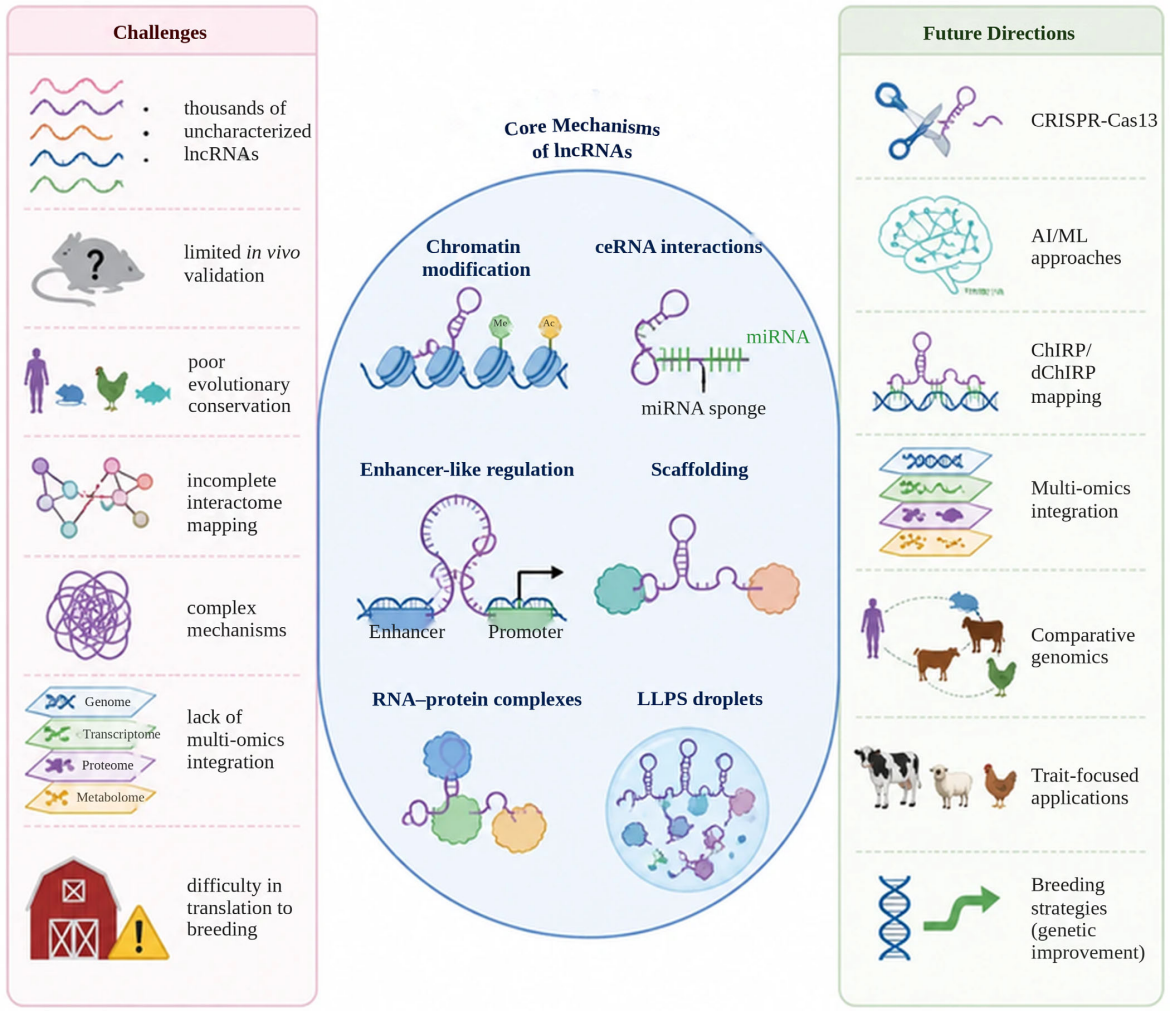
Overview of the major challenges and future directions in lncRNA research in livestock species.

## Author Contributions

K.K., T.H. and R.S. conceived and designed the review framework. K.K. conducted the literature search, performed the analysis and synthesis of information and prepared the original draft. T.H. and R.S. critically reviewed, revised and edited the manuscript. All authors read and approved the final version of the manuscript.

## Funding

The authors have nothing to report.

## Conflicts of Interest

The authors declare no conflicts of interest.

## Ethics Statement

This article is a review based exclusively on previously published studies and publicly available data. No experiments involving animals or humans were conducted by the authors. Therefore, ethical approval and informed consent were not required for this study.

## Data Availability

This article is a review and does not report original research data. All information discussed in this review is derived from cited published literature and publicly accessible databases, including NONCODE, LNCipedia, RNAcentral, Ensembl, NCBI RefSeq/GenBank, and other resources listed in Table 1.
